# Supplementary material for: Monitoring Gene Expression during a Galleria mellonella Bacterial Infection
Source: Microorganisms. 2020 Nov 16;8(11):1798. doi: 10.3390/microorganisms8111798 (PMC7697238; doi:10.3390/microorganisms8111798)

Table S1. Bacterial strains and plasmids used in this study. For each element, a general description is provided, together with an alternative self-explanatory name which will be commonly used in figures to make interpretation of the data easier for the reader. Throughout all the paper, a "P" before the name of a gene indicates the promoter controlling this gene (e.g., P*nrdA* for *nrdAB* operon promoter).

Name	Reference name	Description	Source
**Plasmids**			
pMDM513	pMDM513	Mini-Tn7-GW-Gm derivative vector carrying *luxCDABE*; Amp^R, Gm^R	[1]
pBAM-Gm	pBAM-Gm	pBAM derivative carrying Gm^R	[2]
pJET1.2/blunt	pJET1.2	Blunt-end vector; Amp^R	Thermo Scientific
pETS130-GFP	pETS130	Broad host range, promoterless GFP (mut3-gfp variant); Gm^R	[3]
pETS220-BIATlux	pETSlux	Broad host range, promoterless *luxCDABE*; Gm^R	This work
pETS221	P*nrdA*-lux	pETS220 derivative carrying *nrdA* promoter; Gm^R	This work
pETS222	P*nrdJ*-lux	pETS220 derivative carrying *nrdJ* promoter; Gm^R	This work
pETS223	P*nrdD*-lux	pETS220 derivative carrying *nrdD* promoter; Gm^R	This work
pETS224	P*nrdR*-lux	pETS220 derivative carrying *nrdR* promoter; Gm^R	This work
pETS225	*Anr*-lux	pETS220 derivative carrying *anr* gene fragment; Gm^R	This work
pETS134	P*nrdA*-GFP	pETS130 derivative carrying *nrdA* promoter; Gm^R	[3]
pETS180	P*nrdJ*-GFP	pETS130 derivative carrying *nrdJ* promoter; Gm^R	[4]
pETS226	P*nrdA*-E2Crimson	pETS130 derivative carrying *nrdA* promoter; Gm^R	Lab strain
pETS227	P*nrdJ*-E2Crimson	pETS130 derivative carrying *nrdJ* promoter; Gm^R	Lab strain
**Strains**			
E. coli			
DH5α	DH5α	*recA1 endA1 hsdR17 supE44 thi-1 relA1 Δ(lacZYA-argF)U169 deoR Φ80dlacZM15*	Lab strain
S17.1	S17.1	*recA thi pro hsdR- M+RP4::2-Tc::Mu::Km Tn7 Tpr Smr Xpir*	[5]
P. aeruginosa			
PAO1	PAO1 WT	Wild-type (ATCC 15692/CECT 4122) - Spanish Type Culture Collection	Lab strain
MK171	PAO1::eGFP	*P. aeruginosa* PAO1::mini-Tn7eGFP, Gm^R	[6]
PAO1::lux	PAO1::lux	*P. aeruginosa* PAO1::mini-Tn7l*uxCDABE*, Gm^R	Lab strain

1. Moir, D.T.; Ming, D.; Opperman, T.; Schweizer, H.P.; Bowlin, T.L. A high-throughput, homogeneous, bioluminescent assay for *Pseudomonas aeruginosa* gyrase inhibitors and other DNA-damaging agents. *J Biomol Screen* **2007**, *12*, 855-864, doi:10.1177/1087057107304729.
2. Crespo, A.; Gavalda, J.; Julian, E.; Torrents, E. A single point mutation in class III ribonucleotide reductase promoter renders *Pseudomonas aeruginosa* PAO1 inefficient for anaerobic growth and infection. *Sci Rep* **2017**, *7*, 13350, doi:10.1038/s41598-017-14051-2.
3. Sjöberg, B.M.; Torrents, E. Shift in ribonucleotide reductase gene expression in *Pseudomonas aeruginosa* during infection. *Infect Immun* **2011**, *79*, 2663-2669, doi:10.1128/IAI.01212-10.
4. Crespo, A.; Pedraz, L.; Torrents, E. Function of the *Pseudomonas aeruginosa* NrdR Transcription Factor: Global Transcriptomic Analysis and Its Role on Ribonucleotide Reductase Gene Expression. *PLoS One* **2015**, *10*, e0123571, doi:10.1371/journal.pone.0123571.
5. de Lorenzo, V.; Cases, I.; Herrero, M.; Timmis, K.N. Early and late responses of TOL promoters to pathway inducers: identification of postexponential promoters in Pseudomonas putida with lacZ-tet bicistronic reporters. *J Bacteriol* **1993**, *175*, 6902-6907, doi:10.1128/jb.175.21.6902-6907.1993.
6. Klausen, M.; Heydorn, A.; Ragas, P.; Lambertsen, L.; Aaes-Jørgensen, A.; Molin, S.; Tolker-Nielsen, T. Biofilm formation by *Pseudomonas aeruginosa* wild type, flagella and type IV pili mutants. *Mol Microbiol* **2003**, *48*, 1511-1524, doi:10.1046/j.1365-2958.2003.03525.x.

Table S2. Primers used in this study.

Name	Sequence (5' → 3′)	Application
Lux_SmaI_fw	CCCGGGCATTAAATGGATGGCAAATAT	Cloning
pBAM-Gm up	ACGAACCGAACAGGCTTATG	Cloning
pJET 1.2 fw	CGACTCACTATAGGGAGAGCGGC	Check cloning/Sequencing
pJET 1.2 rv	AAGAACATCGATTTTCCATGGCAG	Check cloning/Sequencing
pBBR1 up	CATCGCAGTCGGCCTATTGG	Check cloning/Sequencing
LuxC_rv	ACCAAGTTCATTTTCTACAACATCA	Check cloning/Sequencing
PnrdA SmaI GFP low	ACCCGGGTTCTCGCGTGTGGTGTCG	P*nrdA* promoter cloning
PnrdA BamHI EcoRI GFP up	AGGATCCGAATTCTTGCTCCACACAGCCTC	P*nrdA* promoter cloning
PJ_SacI up	GAGCTCGGTCCGGCCTGCATCTTC	P*nrdJ* promoter cloning
PnrdJ_SmaI_Rv	CCCGGGGACTGCGTGCGTCTGTC	P*nrdJ* promoter cloning
PD SacI up	GAGCTCCCCGCCTCGCCCAGG	P*nrdD* promoter cloning
PD EcoRI low	GAATTCTTCAACTTCTCCACAACATGAT	P*nrdD* promoter cloning
PR SacI up	GAGCTCCAGGAGAAGGACGGCCAG	P*nrdR* promoter cloning
PR EcoRI low	GAATTCCACCGCAGAAGGGACAAT	P*nrdR* promoter cloning
Anr SacI up_2	GAGCTCTGTTCCGCCAGGGTGA	*Anr* cloning
Anr EcoRI lw	GAATTCTCTTCTTCGACAGCAGCAG	*Anr* cloning
greenQRT_PAO-nrdA_fw	ACCTGGAGAAACTGGGCAAG	qRT-PCR; *P. aeruginosa* RNR genes
greenQRT_PAO-nrdA_rv	TGTGGATGAAGTAGCGGTCG	qRT-PCR; *P. aeruginosa* RNR genes
greenQRT_PAO-nrdJa_fw	CGAATTCATCCGCGCCAAG	qRT-PCR; *P. aeruginosa* RNR genes
greenQRT_PAO-nrdJa_rv	TCCACCGCCTGCATGAAC	qRT-PCR; *P. aeruginosa* RNR genes
greenQRT_PAO-nrdD_fw2	TTGCTGAACGAAGGCCTGAA	qRT-PCR; *P. aeruginosa* RNR genes
greenQRT_PAO-nrdD_rv2	TGCCGAGGAAGTTGACCATC	qRT-PCR; *P. aeruginosa* RNR genes
greenQRT_PAO-nrdR_fw	AACGCTTCACCACCTTCGAG	qRT-PCR; *P. aeruginosa* RNR genes
greenQRT_PAO-nrdR_rv	GCAGCTTGTCCTCGTCGAAC	qRT-PCR; *P. aeruginosa* RNR genes
greenQRT_PAO-norC_fw	AGGGCTTCAACACCTTCCTC	qRT-PCR; *P. aeruginosa* control genes
greenQRT_PAO-norC_rv	CCTCGCTGAGATGGAACTGC	qRT-PCR; *P. aeruginosa* control genes
greenQRT_PAO-gapA_fw	CCTCCCATCGGATCGTCTC	qRT-PCR; *P. aeruginosa* control genes
greenQRT_PAO-gapA_rv	GGTCATCAGGCCGTGCTC	qRT-PCR; *P. aeruginosa* control genes

Table S3. RNA quality. RNA quality parameters obtained from Nanodrop™ analysis for PAO1 in *G. mellonella* infection and in planktonic LB culture for two independent experiments.

Sample	A260	A280	A260/A280	Concentration (ng/µl)
PAO1 in *G. mellonella* Experiment #1	0.258	0.130	1.98	206.72
	0.252	0.128	1.97	201.68
PAO1 in LB Experiment #1	0.272	0.145	1.98	230.08
	0.326	0.172	1.99	248.24
PAO1 in *G. mellonella* Experiment #2	0.078	0.038	2.03	62.56
	0.081	0.041	2.00	65.12
PAO1 in LB Experiment #2	0.123	0.061	2.00	98.16
	0.125	0.062	2.01	99.84

Figure S1. qRT-PCR melting curves. Melting curve analysis for each of the genes analyzed in two independent experiments. Each figure reflects the combined curves of both PAO1 in *G. mellonella* infection and in planktonic culture.

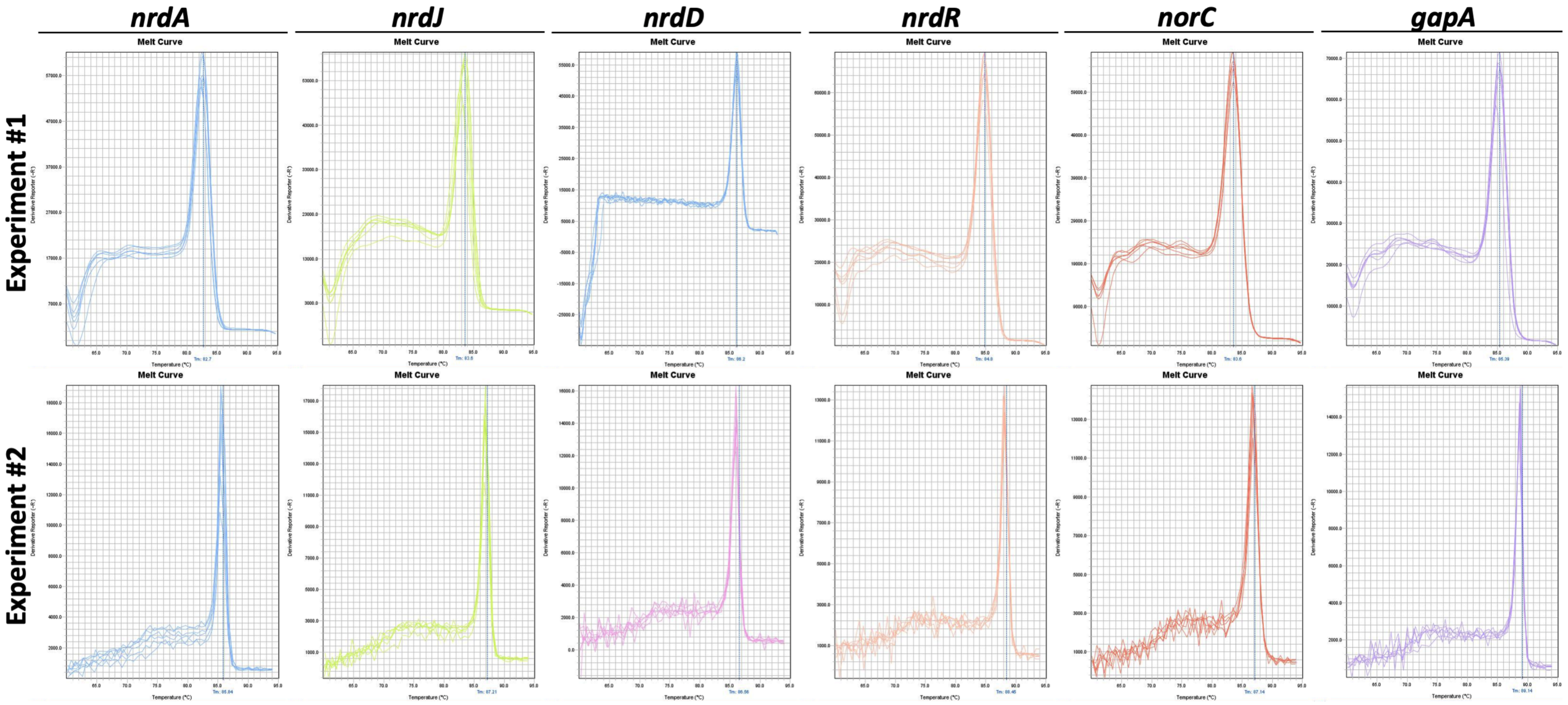

Supplement: Supplementary file 1 [file microorganisms-08-01798-s001.pdf]
